# Supplementary material for: NETosis and Nucleosome Biomarkers in Septic Shock and Critical COVID-19 Patients: An Observational Study
Source: Biomolecules. 2022 Jul 27;12(8):1038. doi: 10.3390/biom12081038 (PMC9405965; doi:10.3390/biom12081038)

biomolecules

MDPI

Supplementary Material

1 Supplementary Figures and Tables

Supplementary Table S1: Demography of the population. Differences between the groups were assessed using a Chi-Square test.

	Control n=48	COVID-19 n=22	Sepsis n=48	p-value
**Demographics**				
Men – n (%)	26 (54)	15 (68)	24 (50)	0.36
Women – n (%)	22 (46)	7 (32)	24 (50)	0.36
Age, years – mean ± sd	61.9±14.5	59.9±10.3	65.0±14.2	0.53
**Medical History**				
Hypertension – n (%)	20 (42)	12 (56)	25 (52)	0.48
BMI > 25 – n (%)	26 (58)	14 (74)	26 (54)	0.34
Diabetes – n (%)	11 (23)	8 (36)	5 (10)	0.71
History of smoking – n (%)	10 (21)	1 (5)	15 (31)	0.04
COPD – n (%)	4 (8)	3 (14)	5 (10)	0.75
CKD – n (%)	9 (19)	0 (0)	10 (21)	0.07
Cancer – n (%)	15 (31)	0 (0)	9 (19)	0.01
**Outcome**				
30-day mortality– n (%)	Not applicable	6 (27)	22 (46)	0.45
ICU length of stay (days) – mean ± SD		29±30	8±9	<0.01
Thromboembolic events – n (%)		6 (27)	4 (8)	0.06
TIMI major bleeding events – n (%)†		5 (23)	1 (2)	0.01
**ICU admission**				
Delays since symptoms (days) – mean ± SD	Not applicable	7.3±3.2	2.6±2.4	<0.01
**Routine laboratory testing**				
Highest CRP (mg/dL) – mean ± SD	Not reported	323±119	313±122	0.75
Creatinine (mg/dL) – mean ± SD		0.91±0.59	2.19±1.91	<0.0
Hemoglobin (g/dL) – mean ± SD		11.62±1.90	10.34±2.05	0.02
Lowest Lymphocytes (10^3/μL) – mean ± SD		484±335	469±310	0.86
**Organ failure and severity scores**				
PaO_2/FiO_2 – mean ± SD	Not applicable	103±37	225±119	<0.01
Ventilation duration (days) – mean ± SD		27±24	4±7	<0.01
Norepinephrine (μg/kg/min) – mean ± SD		0.049±0.105	0.330±0.350	<0.01
Norepinephrine duration (days) – mean ± SD		1.2±3.4	4.8±6.1	<0.01
Renal replacement therapy – n (%)		1 (5)	13 (27)	0.04
Apache II score – mean ± SD		15 ± 4	20 ± 7	<0.01
SOFA Score – mean ± SD		4 ± 1	9 ± 3	<0.01
SIC score – n (%)		0 (0)	11 (24)	0.01
DIC score – n (%)		0 (0)	7 (16)	0.09

†Major bleeding complications have been defined according to the TIMI definition. All bleeding complications in COVID-19 group occurred in ECMO-treated patients.

Abbreviations: *APACHE, acute physiology and chronic health evaluation; BMI, body mass index; COPD, chronic obstructive pulmonary disease; CKD, chronic kidney disease; CRP, C-reactive protein; DIC, disseminated intravascular coagulopathy; ICU, intensive care unit;* PaO_2/FiO_2*, arterial oxygen partial pressure/fractional inspired oxygen; SIC, sepsis-induced coagulopathy; SOFA, sepsis-related organ failure assessment; TIMI, Thrombolysis in Myocardial Infarction; VV ECMO, venovenous extracorporeal membrane oxygenation*

Supplementary Table S2: Summary of biomarkers data in controls, septic shock, and critical COVID-19 subjects. Results are presented as the median (10th – 90th percentile) and the p-value represents the difference between sepsis and COVID-19 patients. Significant results appear in bold characters.

	Parameter	Median (10th - 90th percentile)			
		Control	Sepsis	COVID-19	p-value‡
Circulating nucleosomes and neutrophil activation markers	**[Nu.H3.1] ng/mL**	***24.6 (12.2 - 61.7)***	**862 (253 - 9398)**	**2533 (706 - 4389)**	**0.0020**
	[Nu.Cit-H3R8] ng/mL	*2.46 (1.02 - 7.07)*	71.7 (15.6 - 357)	60.6 (20.8 - 207)	0.2519
	[Cit-H3] ng/mL	*0.215 (0.0757 - 0.679)*	2.82 (0.879 - 13.5)	2.96 (0.748 - 7.72)	0.3786
	[MPO] ng/mL	*23.6 (11.9 - 47.5)*	228 (72 - 2622)	327 (54.7 - 914)	0.6040
	**[NE] ng/mL**	***7.57 (4.13 - 12.6)***	**102 (41.8 - 478)**	**57.2 (20.4 - 178)**	**0.0002**
Pro-inflammatory	**[IL-1β] pg/mL**	***4.00 (3.37 - 4.83)***	**3.71 (1.57 - 7.82)**	**6.74 (4.16 - 10.6)**	**0.0010**
	**[IL-6] pg/mL**	***1.88 (1.32 - 5.01)***	**467 (20.2 - 8199)**	**32.6 (9.07 - 209)**	**<0.0001**
	**[IL-17] pg/mL**	***24.3 (15.3 - 33.9)***	**22.9 (10.9 - 44.8)**	**38.6 (26.8 - 55.8)**	**0.0074**
	**[IFN-ɣ] pg/mL**	***9.15 (3.62 - 18.6)***	**76.5 (12.1 - 249)**	**36.3 (17.3 - 90.8)**	**0.0053**
	**[sTREM-1] ng/mL**	***90.7 (56.8 - 225)***	**450 (158 - 1126)**	**174 (106 - 486)**	**<0.0001**
Anti-inflammatory	**[IL-1ra] pg/mL**	***117 (72.8 - 203)***	**6697 (996 - 18154)**	**645 (235 - 4861)**	**<0.0001**
	**[IL-10] pg/mL**	***4.29 (3.08 - 12.9)***	**18.8 (3.28 - 424)**	**15 (9.68 - 26.8)**	**0.0029**
	**[IL-13] pg/mL**	***2.46 (1.3 - 5.65)***	**1.49 (1.36 - 3.8)**	**4.99 (3.43 - 10.3)**	**<0.0001**
Growth and differentiation factors	[FGF basic] pg/mL	*37.9 (27.2 - 47.6)*	45.8 (26.4 - 71)	60.5 (41.8 - 89.6)	0.1286
	**[G-CSF] pg/mL**	***69.4 (24.7 - 110)***	**268 (62.4 - 18047)**	**98.1 (71.3 - 169)**	**<0.0001**
	[GM-CSF] pg/mL	*1.48 (1.48 - 6.34)*	1.92 (1.56 - 3.55)	1.56 (1.48 - 5.66)	0.8451
	**[IL-2] pg/mL**	***6.52 (6.52 - 13.8)***	**7.24 (7.04 - 19.1)**	**18.3 (9.3 - 33.5)**	**0.0013**
	[IL-4] pg/mL	*3.4 (2.33 - 4.44)*	3.14 (1.64 - 4.74)	4.47 (3.25 - 6.08)	**0.0441**
	**[IL-5] pg/mL**	***80.3 (24.8 - 80.3)***	**20.7 (17.9 - 65.8)**	**37.9 (17.9 - 97.2)**	**0.0130**
	**[IL-7] pg/mL**	***42.3 (28.8 - 61.1)***	**36.7 (17.1 - 62.9)**	**69.1 (44 - 92.2)**	**0.0005**
	[IL-9] pg/mL	*298 (231 - 365)*	178 (132 - 230)	226 (181 - 321)	0.1348
	[IL-12 (p70) pg/mL	*7.28 (7.28 - 7.68)*	8 (7.48 - 12.4)	12.4 (3.52 - 23.7)	0.1822
	[IL-15] pg/mL	*67.6 (67.6 - 77.9)*	63.5 (58.2 - 63.5)	58.2 (58.2 - 103)	0.6641
	**[PDGF-BB] pg/mL**	***285 (188 - 752)***	**98.6 (42.9 - 450)**	**340 (89.7 - 625)**	**<0.0001**
	[TNF-α] pg/mL	*67.3 (51.9 - 89.9)*	93.1 (50.7 - 232)	83.7 (64.2 - 104)	0.1512
	**[VEGF] pg/mL**	***52.7 (52.7 - 64.9)***	**68 (19.7 - 68)**	**19.7 (19.7 - 52.7)**	**0.0002**
Chemoattractant	[Eotaxin] pg/mL	*63.1 (42.3 - 99.4)*	44.1 (21.1 - 117)	40 (24.1 - 63.1)	0.3468
	[ICAM-1] pg/mL	*448 (258 - 682)*	964 (602 - 1972)	868 (527 - 1316)	0.1654
	**[IL-8] pg/mL**	***7.37 (3.4 - 17.5)***	**65 (18.4 - 620)**	**30.7 (15.3 - 49.9)**	**<0.0001**
	**[IP-10] pg/mL**	***520 (274 - 859)***	**1421 (302 - 16973)**	**3487 (985 - 6367)**	**0.0080**
	[MCP-1 (MCAF)] pg/mL	*27.5 (18.5 - 38)*	85.6 (27.6 - 902)	79 (30.6 - 273)	0.0803
	**[MIP-1α] pg/mL**	*1.25 (0.32 - 1.53)*	**7.91 (2.65 - 32.5)**	**3.72 (1.86 - 6.9)**	**<0.0001**
	[MIP-1β] pg/mL	*235 (196 - 266)*	188 (140 - 305)	200 (169 - 231)	0.7722
	[RANTES] pg/mL	*3.5 (1.49 - 7.41)*	2.05 (0.939 - 5.38)	2.13 (0.999 - 3.84)	0.9711
	**[VCAM-1] pg/mL**	***1.07 (0.623 - 2.35)***	**3.37 (1.39 - 4.84)**	**1.71 (1.15 - 4.36)**	**0.0263**
Hemostasis	**[soluble TF] ng/mL**	***46.7 (31 - 74.2)***	**61.3 (25.1 - 108)**	**102 (32.4 - 185)**	**0.0257**
	[TFPI] pg/mL	*20.8 (15.2 - 36.3)*	38.6 (23.2 - 171)	55.9 (19.6 - 80.5)	0.6381
	**[PAI-1] ng/mL**	***28 (12.8 - 61)***	**97.5 (40.6 - 191)**	**64 (37.3 - 78.1)**	**0.0282**
	**[t-PA] ng/mL**	***3.49 (1.85 - 6.08)***	**13.6 (6.5 - 52.4)**	**10.3 (6.34 - 22.6)**	**0.0234**
	**[sTLT-1] ng/mL**	***0.679 (0.0482 - 7.18)***	**1.24 (0.356 - 6.5)**	**0.654 (0.127 - 1.35)**	**<0.0001**
	[sCD62P] ng/mL	*39.5 (27.8 - 56.1)*	58.5 (38.9 - 112)	47.8 (25.4 - 87.6)	0.1226
Cells	**Platelets 10^3/µL**	***241 (162 - 357)***	**180 (50.4 - 409)**	**256 (119 - 443)**	**0.0241**

	**Neutrophils $10^3/\mu L$**	***4.34 (2.37 - 6.35)***	**14.2 (5.11 - 29.9)**	**7.61 (4 - 12.3)**	**0.0025**
	Lymphocytes $10^3/\mu L$	*1905 (1022 - 2967)*	585 (202 - 1444)	910 (451 - 1297)	0.0686

‡ Comparison between sepsis and COVID-19 groups has been done according to an ordinary two-way ANOVA with uncorrected Fischer's significant difference multiple comparison on log-transformed data.

***Abbreviations**: A full list of abbreviation is available in supplementary table S3*

Supplementary Table S3: List of abbreviations of cytokines, hemostasis, nucleosomes and neutrophil activation markers.

Abbreviation	Name of the biomarker
CCL-11	Eotaxin
Cit-H3 (R2, R8 and R17)	Citrullinated histone H3
FGF basic	Basic fibroblast growth factor
G-CSF	Granulocyte colony-stimulating factor
GM-CSF	Granulocyte-macrophage colony-stimulating factor
ICAM	Intercellular adhesion molecule
IL-10	Interleukin 10
IL-12 (p70)	Interleukin 12
IL-13	Interleukin 13
IL-15	Interleukin 15
IL-17	Interleukin 17
IL-1ra	Interleukin 1 receptor antagonist
IL-1β	Interleukin 1 β
IL-2	Interleukin 2
IL-4	Interleukin 4
IL-5	Interleukin 5
IL-6	Interleukin 6
IL-7	Interleukin 7
IL-8	Interleukin 8
IL-9	Interleukin 9
INF-ɣ	Interferon ɣ
IP-10	Interferon γ induced protein 10
MCP-1	Monocyte chemoattractant protein 1
MIP-1α	Macrophage Inflammatory Protein-1 α
MIP-1β	Macrophage Inflammatory Protein-1 β
MPO	Myeloperoxidase
NE	Neutrophil elastase
Nu.H3.1	H3.1-Nucleosome
Nu.H3R8	Citrullinated histone H3R8-Nucleosome
PAI-1	Plasminogen activator inhibitor type 1
PDGF-bb	Platelet-derived growth factor BB
RANTES	Regulated upon Activation, Normal T Cell Expressed and Presumably Secreted
sCD62P	Soluble P-selectin
Soluble TF	Soluble tissue factor
sTLT-1	Soluble TREM-like transcript-1
sTREM-1	Triggering receptor expressed on myeloid cells-1
t-PA	Tissue plasminogen activator
TFPI	Tissue factor pathway inhibitor
TNF-α	Tumor necrosis factor α
VCAM	Vascular cell adhesion molecule
VEGF	Vascular endothelial growth factor

Supplementary Figure S1: Heatmap of cytokine, hemostasis, nucleosome and neutrophil activation markers in controls, septic shock and critical COVID-19 patients.

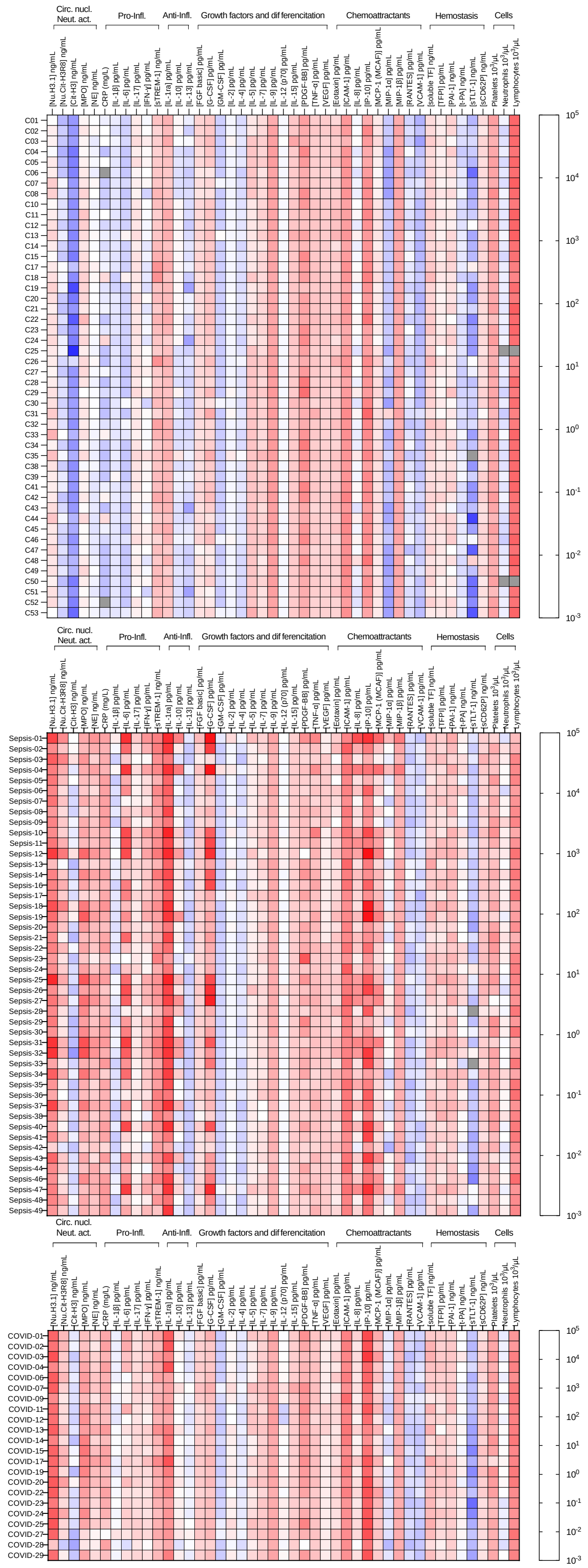

Supplementary Figure S2: Correlation matrix of all parameters reported in this study in controls, septic shock and critical COVID-19 cohorts. Parameters showing a correlation above 0.7 in both cohorts (septic shock and critical COVID-19 patients) are marked in bold. No correlations below – 0.7 were found.

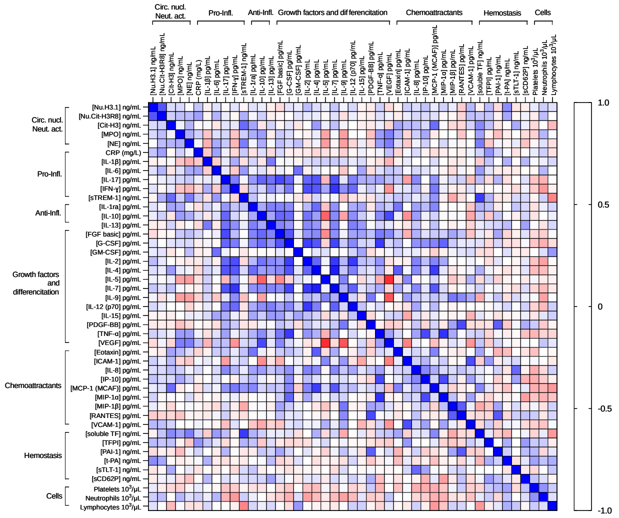

Parameters with Pearson's r > 0.7 or < - 0.7

Controls: Positive correlations

- IL-17 and FGF basic
- INF-γ and IL-7
- TREM-1 and soluble-TF
- G-CSF and IL-2
- G-CSF and IL-7
- G-CSF and TNF-α
- IL-2 and IL-7
- IL-2 and IL-12(p70)
- IL-4 and IL-7
- IL-4 and MCP-1
- PDGF-BB and RANTES
- TNF-α and IP-10
- TNF-α and MIP-1α
- IP-10 and MIP-1α

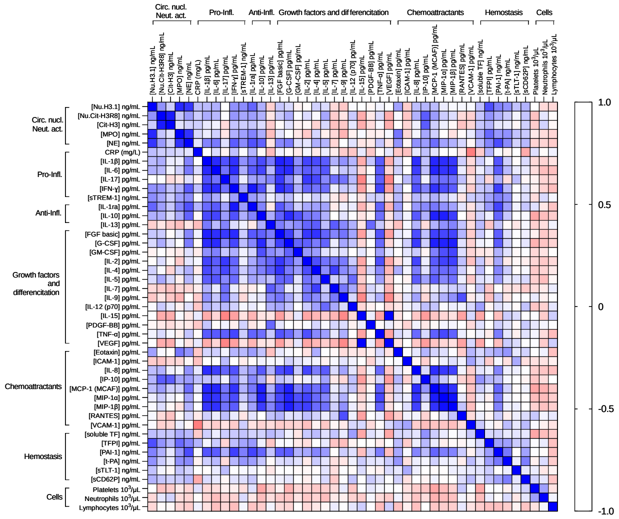

Sepsis: Positive correlations

- Nu.H3.1 and MPO
- NU.H3.1 and NE
- **Nu.Cit-H3R8 and Cit-H3**
- MPO and NE
- NE and PAI-1
- IL-1β and IL-6
- IL-1β and IFN-γ
- IL-1β and IL-10
- IL-1β and FGF basic
- IL-1β and IL-2
- IL-1β and IL-4
- IL-1β and IL-8
- IL-1β and MCP-1
- IL-1β and MIP-1α
- IL-1β and MIP-1β
- IL-6 and INF-γ
- IL-6 and IL-10
- IL-6 and FGF basic
- IL-6 and G-CSF
- IL-6 and IL-8
- IL-6 and MCP-1
- IL-6 and MIP-1α
- IL-6 and MIP-1β
- **IL-17 and FGF basic**
- **IL-17 and IL-2**
- **IL-17 and IL-4**
- **INF-γ and IL-ra**
- INF-γ and FGF basic
- INF-γ and G-CSF
- INF-γ and TNF-α
- **INF-γ and MIP-1α**
- INF-γ and MIP-1β
- INF-γ and PAI-1
- IL-1ra and PAI-1
- IL-10 and MCP-1
- IL-10 and MIP-1α
- IL-10 and MIP-1β
- **FGF basic and IL-2**
- **FGF basic and IL-4**
- FGF basic and MCP-1
- FGF basic and MIP-1α
- FGF basic and MIP-1β
- G-CSF and IL-8
- G-CSF and MCP-1
- G-CSF and MIP-1α
- G-CSF and MIP-1β
- GM-CSF and MIP-1β
- IL-2 and IL-8
- IL-2 and MIP-1α
- IL-2 and MIP-1β
- **IL-4 and IL-7**
- TNF-α and MIP-1α
- IL-8 and MCP-1
- **IL-8 and MIP-1α**
- IL-8 and MIP-1β
- MCP-1 and MIP-1α
- MCP-1 and MIP-1β
- MIP-1α and MIP-1β

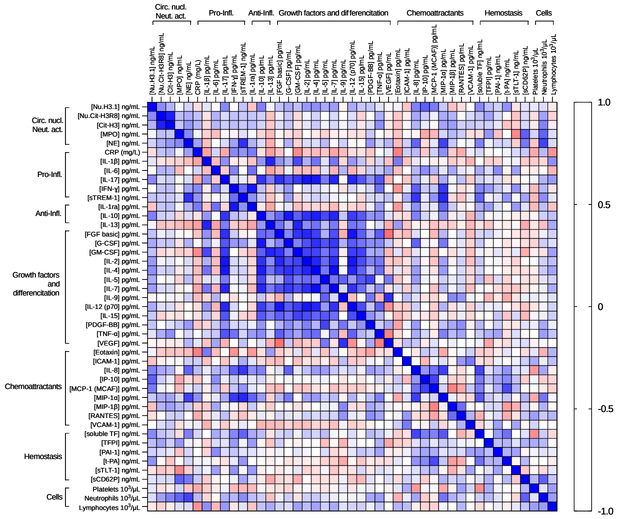

COVID-19: Positive correlations

- **Nu.Cit-H3R8 and Cit-H3**
- NE and sTREM-1
- NE and MIP-1α
- NE and neutrophils
- IL-1β and IL-13
- IL-1β and GM-CSF
- IL-17 and IL-10
- **IL-17 and FGF basic**
- IL-17 and GM-CSF
- **IL-17 and IL-2**
- **IL-17 and IL-4**
- IL-17 and IL-7
- IL-17 and IL-12(p70)
- **INF-γ and IL-1ra**
- **INF-γ and MIP-1α**
- sTREM-1 and IL-8
- sTREM-1 and MIP-1α
- IL-10 and G-CSF
- IL-10 and IL-2
- IL-10 and IL-4
- IL-10 and IL-5
- IL-10 and IL-7
- IL-10 and IL-12(p70)
- IL-13 and GM-CSF
- FGF basic and GM-CSF
- **FGF basic and IL-2**
- **FGF basic and IL-4**
- FGF basic and IL-12(p70)
- G-CSF and IL-4
- G-CSF and IL-7
- GM-CSF and IL-2
- GM-CSF and IL-4
- GM-CSF and IL-12(p70)
- GM-CSF and IL-15
- IL-2 and IL-4
- IL-2 and IL-7
- IL-2 and IL-12(p70)
- IL-2 and IL-15
- **IL-4 and IL-7**
- IL-4 and IL-12(p70)
- IL-5 and PDGF-BB
- IL-7 and IL-12(p70)
- IL-12(p70) and IL-15
- **IL-8 and MIP-1α**
- IL-8 and soluble TF
- IP-10 and MCP-1
- MCP-1 and t-PA

Supplementary Figure S3: Correlation between Nu.H3.1 and APACHE-II and SOFA scores in septic shock and critical COVID-19 patients.

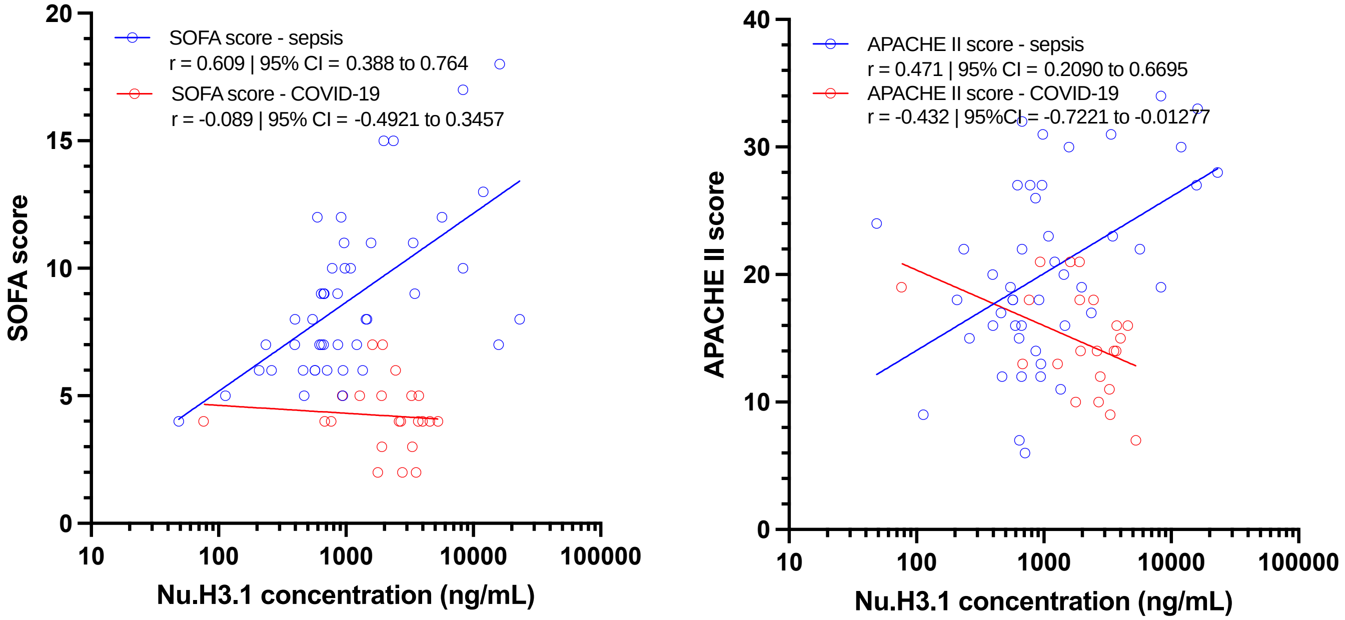

Supplementary Figure S4: Correlation between NE and Nu.H3.1 in septic shock and critical COVID-19 patients.

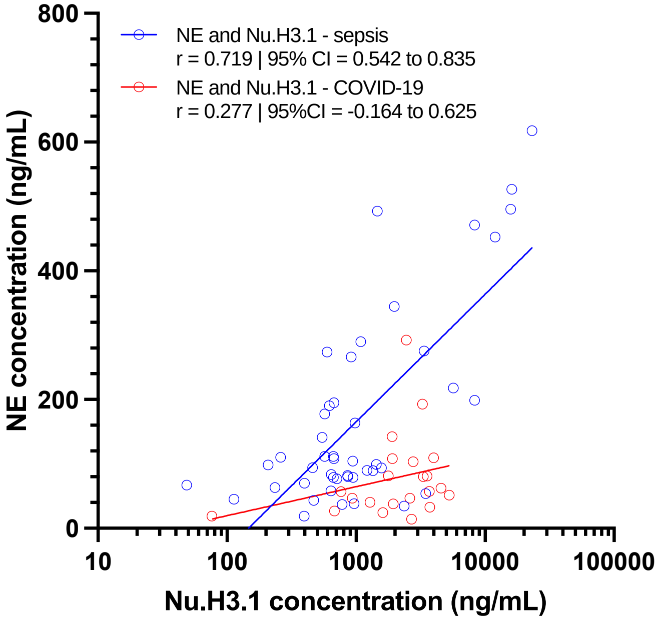

Supplement: Supplementary file 1 [file biomolecules-12-01038-s001.zip › biomolecules-1761164-supplementary.pdf]
